# Impact of gender and primary tumor location on outcome of patients with cutaneous melanoma

**Published:** 2016

**Authors:** S Voinea, A Blidaru, E Panaitescu, A Sandru

**Affiliations:** * Department of Surgical Oncology, “Carol Davila” University of Medicine and Pharmacy; “AlexandruTrestioreanu” Oncologic Institute, Bucharest, Romania; ** Department of Medical Informatics and Biostatistics, “Carol Davila” University of Medicine and Pharmacy, Romania; *** Department of Surgical Oncology, “Alexandru Trestioreanu” Oncologic Institute, Bucharest, Romania

**Keywords:** malignant melanoma, patient gender, primary tumor location, disease free survival, overall survival

## Abstract

**Background.** The survival of patients with cutaneous malignant melanoma (MM) depends on multiple factors whose role is continuously updated, as the molecular mechanisms underlying the disease progression are understood. This study intended to assess whether the patient’s gender and tumor location affect the disease outcome.

**Methods.** Between 2008 and 2012, 155 patients with cutaneous MM underwent various types of surgeries in our clinic. Patients were staged according to the 2009 TNM classification. There were 90 women and 65 men. Primary tumors were located as it follows head and neck region - 4.5%, limbs - 50.7% and trunk - 44.8%. The disease free and overall survival rates (DFS, OS) were estimated by using the Kaplan-Meier method.

**Results.** Metastases developed in 52.3% of the males and 31.1% of the females (p=0.008). In univariate analysis, distant metastasis risk was significantly higher in men (p = 0.0472 for stage II patients and p = 0.0288 for stage III). In multivariate analysis, male gender almost doubled the risk of relapse (p = 0.044) and death (p = 0.022). Consequently, DFS and OS were significantly higher among females. Primary tumor location seemed to influence the melanoma spreading ability. Half of the trunk MM developed metastases while only a third of limbs MM did. The association between MM location and the recurrence risk was not random (p = 0.033).

**Conclusions.** The patient gender represents an independent prognostic factor for both relapse and death. Although trunk MM had a significantly higher risk of metastasis than limbs MM, the location per se was not an independent prognostic factor for survival (p = 0.078).

**Abbreviations:** MM = malignant melanoma, DFS = disease free survival, OS = overall survival, p = p value, AJCC = American Joint Commission on Cancer, CI = confidence interval

## Introduction

Cancer patients’ survival is influenced by many factors, whose share in the disease progression is difficult to assess. Unquestionably, the disease stage at diagnosis is the parameter with the greatest impact on survival, but often, patients diagnosed in the same clinical stage, having tumors with similar pathological features and undergoing similar therapeutic procedures, have a completely different evolution. Hence, the attempts to provide a personalized treatment adapted to the genetic and biochemical changes specific to each individual tumor.

Although previous statements are valid for any neoplasia, cutaneous malignant melanoma (MM) has perhaps, the most unpredictable evolution, and the attempt to fit it into a pattern often fails.

In this paper, we aimed to investigate whether the patients’ gender and primary tumor location in a particular segment of the body (trunk, upper or lower limbs) affect the prognosis of patients with MM. The influence of these two factors overlaps partly because it seems that the patient’s gender determines, to a certain extent, the MM location [**1**]. 

Several epidemiological studies have described that both MM incidence and survival vary significantly according to the patients’ gender [**2**-**4**]. In Caucasian populations, MM incidence is significantly higher in women than in men, but the negative impact of this finding is mitigated by the fact that for the same clinical stage of the disease, women live longer than men [**2**-**4**].

According to many authors, in metastases free MM patients, the primary tumor location on a particular segment of the body significantly influences survival [**5**,**6**]. Tumors of the limbs have a better prognosis than those developed on the trunk or head and neck region [**5**-**7**], but so far, there has been no scientific explanation for this observation.

Starting from the literature data presented above, we examined whether gender and primary tumor location in our group affect survival or not.

## Materials and Methods

155 patients with MM were surgically treated and followed-up between June 2008 and December 2012, in the 2nd Clinic of Surgical Oncology from “AlexandruTrestioreanu” Oncologic Institute in Bucharest. The diagnosis of MM was established, in most cases (153), by excisional biopsy of the primary tumor. Further treatment varied, depending on the patient’s clinical stage at diagnosis.

Patients were staged according to AJCC 2009 (American Joint Commission on Cancer) as it follows: stage 0 - 2 patients, stage I - 31 patients, stage II – 72 patients, stage III - 47 patients and stage IV – 3 patients.

There were 90 women (58.1%) and 65 men (41.9%) within the cohort. Primarytumors were unevenly distributed throughout the body segments: 7 (4.5%) in the head and neck region, 51 (33.1%) in the lower limbs, 27 (17.5%) in the upper limbs and 69 (44.8%) in the trunk.

After an appropriate treatment, patients were submitted to periodic controls, at every 3 to 6 months (according to the pathological stage), which consisted in a complete physical exam, abdominal and regional lymphatic basin ultrasound and chest radiography. If these routine tests have raised suspicion of metastasis, then the investigations were supplemented with computed tomography and, in a few cases, with positron emission tomography.

The mean follow-up period was of 31 months, with a standard deviation of 27.26 months. Half of the patients were followed for at least 24 months.

SPSS 15.0 system was used for the statistical analysis.Kaplan-Meier analyses with a confidence interval of 95% were performed to estimate and to compare disease free and overall survival. Survival time differences for different gender and tumor locations were analyzed by using the Log Rank, Wilcoxon and Cox tests. Multivariate survival analysis was performed by using Cox logistic regression modeling with a P value of less than 0.05 being considered as statistically significant.

## Results

The risk of developing distant metastases was higher in men, in the MM patients treated in our clinic. Thus, during the study, 52.3% of the males developed distant metastases, while in females this life threatening event occurred in only 31.1% of the cases (p = 0.0078, chi-square test).

Detailing the analysis for each stage separately, we found that the risk of distant metastasis was significantly higher in men than in women, irrespective of the clinical stage at diagnosis (p = 0.0472 for stage II patients and p = 0.0288 for stage III patients).

Regarding the percentage of locoregional recurrences, although it was higher in females than in males, 32.2% versus 23%, the difference did not reach a statistical significance for our group (p = 0.62).

**Fig. 1:**
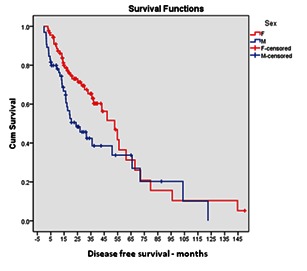
Kaplan-Meier analysis of DFS according to patient gender

Overall, males had a 1.6times higher likelihood of different types of recurrences (regardless of their location), which resulted in a disease free survival (DFS) significantly longer in women (**Fig. 1**). The statement was verified by three different tests of statistical significance (log rank, Wilcoxon and Cox Proportional Hazards) and the conclusion was that the observed differences between the genders’ disease progression were not random (p = 0.0457; 95%CI = 1.009 - 2.537).

All the survival data had higher values in women. The median survival in our group was double in women than in men, 61 months versus 30 months, and the women’s mean survival of 76.5 months was significantly higher than that of men, 55.4 months.

**Fig. 2:**
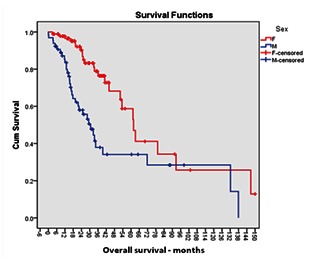
Kaplan-Meier analysis of OS according to patient gender

As shown in **Fig. 2**, the overall survival (OS) in our lot was significantly higher in women than in men (p = 0.004), male gender looming as an independent risk factor for death (HR = 2.625; 95% CI = 1.536 - 4.868). For our group of patients, the death probability of a man with MM was 2.6 times higher than that of a woman with MM (univariate Cox). Consequently, by the end of the study, 52.3% of the men were dead compared to only 28.9% of women.

Another finding of our study was that the location of melanic lesions was influenced by the patients’ sex. Previous remarks depicted that men had an increased risk of developing MM on the trunk, head and neck, while women tended to develop such lesions on the limbs, predominantly the lower ones [**1**,**2**,**8**].

In our analysis, 70.5% of the MM diagnosed in the limbs belonged to women and 57.8% of the MM arising in men were located on the trunk. In other words, while women developed limbs MM at a rate of 61.1%, these segments were interested in males only in 35.9% of the cases.

The difference between the proportions of tumors developed on the trunk in relation to patient gender was not as spectacular as for the limbs, but has retained a statistical significance: 53.6% of the axial MM occurred in males and 46.4% in females. The observed distribution was analyzed by using the Likelihood Ratio Test. Taking into account a p value of 0.0008, we believed that the MM preferential appearance on a certain segment of the body, depending on the patient’s gender, was not accidental.

## Discussions

In European countries, MM occurs in a larger proportion in women than in men (58.1% in our study were women), while in the US and Australia, it developed more frequently in men [**9**]. Unlike incidence, which is different between continents, melanoma survival seems to have the same trend worldwide: women with MM live longer than men, irrespective of latitude or longitude [**2**-**4**]. The question that remains unanswered is which are the mechanisms that lie behind this behavior? What gives women an advantage in the fight against MM? Opinions are divided and contradictory in many respects.

Some studies have concluded that gender correlates with patient prognosis as it determines a certain pattern of metastasis [**2**]. Retrospective analyses on large databases showed that the risk of distant metastasis is lower in women than in men; instead, the likelihood of locoregional relapse is superior in women [**2**]. If the above-mentioned remarks are confirmed, this particularity of MM evolution could also explain the different rates of survival.

By using a multivariate logistic regression that took into account the clinical stage at diagnosis, primary tumor thickness, presence of lymphovascular invasion and patients’ sex, our study revealed that male gender was an independent negative prognostic factor for disease free survival. Men have a risk of relapse 1.7 times higher than women (HR = 1.729; 95%CI = 1.0135 - 2.9497), irrespective of the clinical stage at diagnosis (multivariate logistic regression). Considering the results, we believe that the assumption made by Lasithiotakis et al. [**2**] is checked in our study too (p = 0.0445).

Going through the literature, we found that men have a death probability due to metastatic melanoma 1.9 times higher than women [**9**]. In our study, males had twice the death risk of females (HR = 2.0626; 95%CI = 1.1103 - 3.8316), and this risk has been preserved for each stage separately (p = 0.022).

In a review of 2672 MM patients from the EORTC database, it was found that women were likely to develop regional and distant recurrences by 30% less than men, which translated into a 30% higher OS and DFS [**3**]. In our group, women have developed metastases at a rate by 20% less than men, i.e. 30% versus 50.8%. This difference resulted in a superior women survival: at the end of the study, 71.1% of the women were alive, while the percent in men was of only 47.7%.

Various explanations for these statistical findings have been attempted; most of them focusing on the role of hormones in the disease etiopathogenesis, but the published results are conflicting. Although initially it was assumed that the estrogen hormone might have a protective role, recent data claim that MM is not a hormone-dependent tumor and the clinical trials with antiestrogens had disappointing results [**2**]. On the other hand, women benefit in terms of survival is maintained regardless of reproductive, menstrual (pre- or postmenopausal) and hormonal status [**9**,**10**], suggesting that estrogens are not the ones that provide protection.

Although the mechanism by which patient gender determines significant differences in the disease outcome has not been elucidatedyet, two hypotheses have attempted to explain the phenomenon [**3**,**10**]:

• Behavioral differences between the two sexes (women are more careful about their appearance) lead to a late diagnosis of MM in men with a negative impact on survival (thicker tumors, commonly ulcerated, localized mainly on the trunk). From our point of view, this hypothesis is only partially valid. Indeed, in our study also, the percentage of men with advanced tumors was higher than that of women, but the logistic regression for every clinical stage showed that male gender had a negative impact on OS and DFS, independent of other prognostic factors taken into account.

• The biological differences, yet unproven, between the two genders affect the progression of MM (it is speculated that tumor-host interaction changes according to gender: in males, some metastasis stimulating factors would be secreted, while in women some inhibitor ones). The hypothesis is fascinating, but without consistent evidence yet.

Nowadays, most theories that try to explain the two genders different disease evolution are focused on the demonstration of the second mentioned hypothesis, namely that in women, melanoma cells have a lower potential for dissemination than in men. The ability of progression is diminished in all the phases of the disease: in women, melanocytes have a lower capacity for invasion, survival in the bloodstream, implantation, and colonization in various organs [**4**]. However, these assumptions cannot explain the finding that women survive longer in stage IV, inclusively.

As stage IV contains a wide range of metastases locations with different vital prognoses, Joose et al. considered in their analysis that the milder disease evolution in women with stage IV could be explained by the fact that women have a lower risk of developing visceral metastases, even 50 percent less than men[**4**].

In the group we followed, 88.3% of the metastases identified in males were located in the brain, lung, liver, while in women only 71.4% of distant metastases had a visceral distribution; the remaining metastases have been scattered in extra-regional lymph nodes and subcutaneous tissue, locations with no immediate impact on survival, as we reported in a previous study [**11**]. However, if we compared the number of visceral metastases to the entire cohort of men, respectively women, we would see that 46% of the men developed metastases in vital organs, while only 22.2% of the women presented this pattern. The greater survival of women in stage IV could be explained up to a point by the higher proportion of metastases developed in the peripheral, superficial regions, which allow the early detection of new tumors by patients themselves and an easier surgical approach [**11**]. Nevertheless, this cannot be the only explanation, because there are papers stating that women keep their survival advantage over men even in the case of visceral spread [**4**].

Another parameter credited as an independent prognostic factor for melanoma patients survival is the location of the primary tumor [**7**,**12**,**13**]. However, this factor is, in its turn, closely related to the patient sex, as revealed in our study. Therefore, there is the possibility that primary melanoma location loses its significance in a multivariate analysis.

As several studies have concluded that MM developed on the trunk have a poor prognosis compared to those located in the limbs [**7**,**12**,**13**], we aimed to check this information in our cohort. For this purpose, we have estimated the risk of local recurrence, regional and distant metastasis, as well as risk of death according to primary tumors distribution in various segments of the body.

In the follow-up period, half of the MM occurred metastasized on the trunk (50.7%), while only a third of those were the diagnosed in the limbs (33.3%). Of the 61 patients with distant relapses, 57.4% had a primary melanoma on the trunk. Using Chi-square test, we obtained a p value of 0.0327, which proved that there is a non-random association between the risk of metastasis and the primary tumor location.

We also found that the death rate of patients with MM located in various regions of the trunk was 46.4%. The evolution of limbs tumors was less aggressive, death occurring only in 34.7% of the cases. Although both literature data, as well as those in our survey up to a point, suggested an association between the primary tumor location and death probability, the statistical analysis of the information collected from our patients (Chi-square test) did not allow a definite conclusion in this regard (p = 0.0752).

In our lot, OS and DFS were not significantly different between the group of patients with trunk tumors and those with limbs ones (p = 0.078, respectively p = 0.431). We found a similar conclusion in a French paper, which considered that topography influenced only the primary tumor lymphatic drainage pattern, not the patients’ prognosis [**14**]. However, both the French study, as well as ours, had the same limitation: the small number of cases, which might alter the statistical significance of the observed phenomena.

## Conclusions

It seems that the melanoma natural history is different in women compared to men. Nevertheless, the underlying reason of the milder behavior of cutaneous melanoma in women is still a subject of debate. Instead of conclusion, Wallace Clark’s statement from 1969should be reminded, who believed even since then, that melanoma “is somewhat less malignant in the female when compared with male” [**15**]. Is it possible that women have a biological advantage in the fight against cancer? We cannot definitely state this yet, but the information gathered up to now, converge towards this conclusion.

We hope that further research will find out the biological mechanism of this intriguing evolution.

**Disclosure**

The authors declare no potential conflict of interests.

**Source of founding**

Project PN-II-PCCA type 2 Nr.4/2012 (HRCarrays) “Array structures for prevention, individualized diagnosis and treatment in cancers with high risk of incidence and mortality”.
